# Application of response surface methodology for optimization of decolorization and mineralization of triazo dye Direct Blue 71 by *Pseudomonas aeruginosa*

**DOI:** 10.1007/s13205-013-0192-7

**Published:** 2013-12-29

**Authors:** Maryam Khosravi Hafshejani, Chimezie Jason Ogugbue, Norhashimah Morad

**Affiliations:** 1 Environmental Division, School of Industrial Technology, Universiti Sains Malaysia, Penang, Malaysia; 2 Department of Microbiology, Faculty of Biological Science, College of Natural and Applied Sciences, University of Port Harcourt, Choba, Nigeria

**Keywords:** Response surface methodology, Azo dye, *Pseudomonas aeruginosa*, Decolorization, Degradation

## Abstract

The decolorization and degradation of Direct Blue 71 were investigated using a mono culture of *Pseudomonas aeruginosa*. The bacterium was able to decolorize the dye medium to 70.43 % within 48 h under microaerophilic conditions. The medium was then aerated for 24 h to promote the biodegradation of the aromatic amines generated from azo bond cleavage. Reduction in total organic carbon in dye medium was 42.58 % in the microaerophilic stage and 78.39 % in the aerobic stage. The degradation metabolites formed were studied using UV–vis techniques, high performance liquid chromatography, Fourier transform infra red spectroscopy and nuclear magnetic resonance spectroscopy analysis. Data obtained provide evidence for the formation of aromatic amines and their subsequent oxidative biodegradation by a single strain of *P. aeruginosa* during successive microaerophilic/aerobic stages in the same flask. The influence of incubation temperature (20–45 °C), medium pH (5–10) and initial dye concentration (25–150 mg/L) on decolorization was evaluated to greatly influence decolorization extent. The optimal decolorization conditions were determined by response surface methodology based on three-variable central composite design to obtain maximum decolorization and to determine the significance and interaction effect of the variables on decolorization. The optimal conditions of response were found to be 35.15 °C, pH 8.01 and 49.95 mg/L dye concentration giving an experimental decolorization value of 84.80 %. Very high regression coefficient between the variables and the response (*R*^2^ = 0.9624) indicated a good evaluation of experimental data by polynomial regression model.

## Introduction

The huge growth in the textile dyeing and dyestuff manufacturing industries has resulted in a proportional increase in the volume and complexity of the wastewater discharged to the environment, making it one of the main sources of severe pollution problems worldwide (Maljaei et al. 2009; Cervantes and Dos Santos 2011). Textile industrial effluents often contain a significant amount of residual dye due to the inefficiency in dyeing processes and it is estimated that 5–50 % of unfixed dyes are lost in the effluent during the dyeing process (Maljaei et al. 2009), which ultimately find their way into the environment. Because of their recalcitrance, low biodegradability (ca. BOD_5_/COD <0.2–0.3), toxic nature and the esthetic problems they cause in water bodies, the loss of dyes to the environment may present eco-toxic hazards and introduce the potential danger of bioaccumulation that may eventually affect man by transport through the food chain (Dos Santos et al. 2006).

One of the most widely used of the synthetic dyes, which are usually considered major pollutants in textile wastewater, is the azo dyes (Maljaei et al. 2009). Azo dyes characterized by the presence of one or more azo groups (i.e. the chromophore) are widely used in textile, printing, cosmetics, pharmaceutical, food and many other industries. They consist of over 60–70 % of the global dyestuff production which stands at 3.49 × 10^10^ kg and accounted for annual global sales of nearly US$ 6 billion (Dyes and Pigments 2010). Due to the large degree of aromatics present in the dye molecule and the stability of modern dyes, they are difficult to destroy or decompose by common treatment in conventional wastewater treatment plants (Baughman and Weber 1994; O’Neill et al. 2000). Hence, the textile dyeing industry is under considerable pressure to develop suitable treatment methods to efficiently and effectively treat the effluents discharged to the environment to comply with environmental legislation restricting the discharge of wastewater and to prevent deterioration of ecosystems.

Treatment of azo dye containing wastewater has been carried out using physicochemical methods such as coagulation and flocculation, adsorption, ozonation, photochemical oxidation, membrane filtration and electrochemical oxidation. These methods have earlier been extensively reviewed (Robinson et al. 2001; Forgacs et al. 2004; Joshi et al. 2004). However, these methods remain unattractive for industrial use because of associated limitations such as high cost, limited versatility, interference by other wastewater constituents, secondary pollution problems that may arise due to excessive use of chemicals, and generation of large quantities of sludge that may be difficult to dispose (Stolz 2001; Jadhav et al. 2007). These limitations have spurred renewed search for new treatment methods and have shifted attention to biological methods which are easy to operate, cost effective, eco-friendly and amenable to scale up in the field (He et al. 2004; Rodriguez Couto et al. 2006; Dafale et al. 2008).

Numerous attempts have been made to develop bioprocesses for the treatment of textile effluents using fungi and bacteria. However, the use of bacterial cultures is preferable since they exhibit a rapid growth rate and require shorter hydraulic retention time for dye decolorization. Previous reports indicated that bacterial strains like *Bacillus firmus* (Ogugbue et al. 2012a), *Aeromonas hydrophila* (Ogugbue et al. 2012b), *Klebsiella* sp. strain VN-31 (Franciscon et al. 2009), *Sphingomonas* sp. (Hsueh and Chen 2008) and *Dyella ginsengisoli LA*-*4* (Zhao et al. 2010) had shown very promising results for dye decolorization under anoxic conditions. In most cases, decolorization of the azo dyes was accompanied by the accumulation of toxic, mutagenic and carcinogenic aromatic amines that are recalcitrant to degradation under anoxic conditions, apart from having potentials of bioaccumulating in the food chain (Dos Santos et al. 2006; Isık and Sponza 2008). Hence, along with color removal, total degradation of azo dyes is the only solution for final elimination of these xenobiotics from the environment (Mohana et al. 2008).

Until now, the effects of environmental factors on microbial decolorization of azo dyes are usually examined with the conventional single-factor optimization (Parshetti et al. 2006; Khataee et al. 2009; Sedighi et al. 2009) in which experiments were conducted by varying systematically the studied parameter while keeping other parameters constant. This is usually repeated for all the parameters influencing decolorization thus, resulting in an unreliable number of experiments. In addition, the combined effect of the effective influence parameters cannot be determined using this exhaustive procedure. Hence, a novel experimental design method such as the response surface methodology (RSM) which can estimate linear interaction and quadratic effects of the factors and predict a model for the response with a minimum number of experiments could be a useful tool for optimization of effective parameters of decolorization.

Here, we report the isolation and identification of a novel dye degrading bacterium, *P. aeruginosa*, capable of rapid azo dye decolorization and mineralization of generated intermediate metabolites (aromatic amines) in a sequential microaerophilic and aerobic process, respectively. There are almost no reports on the biodegradation of azo dyes by *P. aeruginosa* and hence, it was pertinent to develop this new microbial resource in environmental bioremediation for azo dye decolorization. The effects of environmental parameters on decolorization were determined and the decolorization conditions optimized using the Response surface methodology (RSM) based on central composite design (CCD).

## Materials and methods

### Dyes, chemicals and culture media

**Fig. 1:**
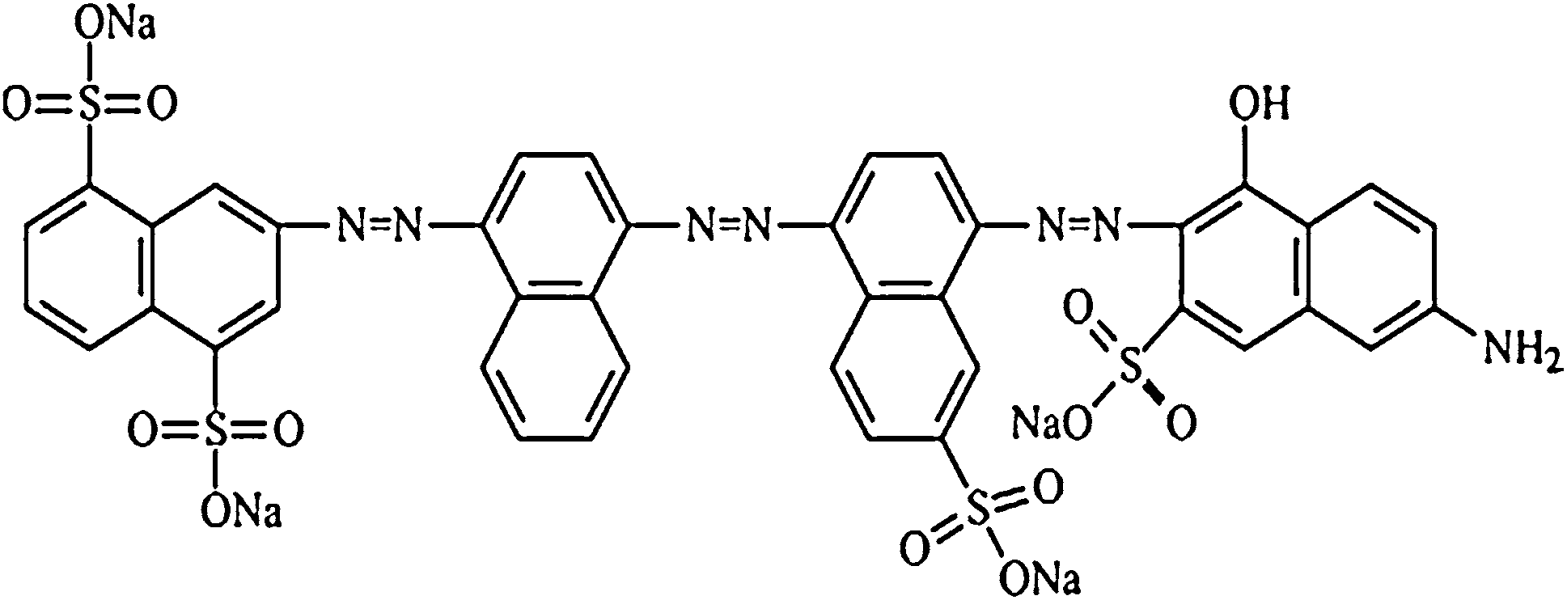
Chemical structure of the model dye, Direct Blue 71 (C.I. 34,140; *λ*_max_ 587 nm), used in this study

Direct Blue 71 (C.I. 34,140; *λ*_max_ 587 nm; dye content, 50 %) used in this study was purchased from Sigma-Aldrich Chemical co. USA. This azo dye was used as received without further purification. Stock solution of the dye was prepared by adding 10 g of dye powder in 1 L of deionized water and sterilized by membrane filtration using a 0.02 μm pore size membrane filter. Figure 1 shows the chemical structure of Direct Blue 71. Other chemicals or reagents used were of analytical grade. Bushnell Haas Medium (in g/L: MgSO_4_, 0.2; K_2_HPO_4_, 1.0; KH_2_PO_4_, 1.0; CaCl_2_, 0.02; FeCl_3_, 0.05; and NH_4_NO_3_, 1.0) was used for isolation of dye degrading bacteria whereas the modified version of the medium which contained glucose (5 % w/v) and yeast extract (0.09 % w/v) was used for decolorization experiments.

### Strain isolation and characterization

The organism (*P. aeruginosa*) used in this study was isolated from textile industrial wastewater using the selective enrichment culture method in Bushnell Haas Medium amended with Methyl Red as previously described (Khosravi et al. 2012). The isolate, selected from a group of dye degrading bacteria obtained, was chosen for achieving the best decolorization and for its broad spectrum azo dye biodegradability potential. The isolate was identified by comparing its 16S rDNA sequence with the 16S rRNA sequence data of reference and type strains obtained from the GenBank databases using the BLASTn as previously described (Khosravi et al. 2013). The organism was used for subsequent decolorization and optimization studies.

### Decolorization of direct Blue 71 by *Pseudomonas aeruginosa*

The decolorization capability of *P. aeruginosa* was determined using the selected azo dye (Direct Blue 71, 50 mg/L) in modified BHM. An activated culture (10 % v/v; OD_660 nm_ 0.6) of the bacterium was inoculated into Erlenmeyer flasks containing 200 mL of pre-autoclaved BHM (yielding approximate cell densities of 10^7^ CFU/mL; pH, 7) and incubated at 30 °C for 48 h under static conditions to achieve microaerophilic conditions. Decolorization was more rapid under microaerophilic conditions with this bacterium from previous experiments (Khosravi et al. 2013). The culture flasks were then further incubated under aerobic conditions for another 24 h making a total incubation time of 72 h. Aerobiosis was to encourage degradation of aromatic amines generated during decolorization due to the cleavage of azo bonds in the first 48 h of incubation. Samples were withdrawn intermittently (every 4 h) during incubation and used for determination of dye decolorization by monitoring the absorbance of clarified samples and to determine the equilibrium time required for maximum dye decolorization. Controls consisted of dye broths maintained without bacterial culture.

Further experiments were performed to determine the effect of incubation temperature, pH and initial dye concentration (dosage) on dye degradation by varying the incubation temperature (20–45 °C), medium pH (5–10) and concentration of the dye in BHM (25–150 mg/L), while keeping other conditions constant. The pH of the BHM solution was adjusted using 0.1 M HCl or 0.1 M NaOH. All the experiments were performed in triplicates.

### Optimization of decolorization conditions by response surface methodology

RSM is a collection of mathematical and numerical techniques that are useful for modeling and analysis of the processes having numerous variables influencing the response and the objective is to optimize process settings in an efficient use of the resources (Sharma et al. 2009). It can be used for predicting the functional relationship between a set of experimental design variables and a response variable. The RSM approach was applied to determine the optimal levels of three input variables, namely temperature (factor A), pH (factor B) and dosage (factor C) and to identify the relationship between the response functions and process variables. Design Expert 6.0.8 software was used to analyze the obtained results. The values used were based on results of preliminary experiments carried out to determine range of values of parameters for effective decolorization.

For three variables (*n* = 3) and five levels [low (−) and high (+)], the total number of experiments was 36 determined by the expression: 2^*n*^ (2^3^ = 8 factorial points in triplicates) + 2*n* (2 × 3 = 6 axial points) + 6 (center points: six replications) as given in Table 1. Dye decolorization was selected as the response for the combination of the independent variables. Randomised experimental runs were performed to reduce to the barest minimum the effects of unexpected variability in the observed responses (Montgomery 2004).

**Table 1:** Observed and predicted values of Direct Blue 71 decolorization by *P. aeruginosa*

Design points	Point type	Coded independent variable levels			% (responses)	
		A	B	C	Experimental (observed value)	Predicted value
1	Fact	30.00	8.00	40.00	64.6517	73.02
2	Fact	30.00	8.00	40.00	78.0224	73.02
3	Fact	30.00	8.00	40.00	77.8223	73.02
4	Fact	40.00	8.00	40.00	72.2578	80.58
5	Fact	40.00	8.00	40.00	88.4307	80.58
6	Fact	40.00	8.00	40.00	86.8695	80.58
7	Fact	30.00	10.00	40.00	54.2834	57.95
8	Fact	30.00	10.00	40.00	59.4075	57.95
9	Fact	30.00	10.00	40.00	62.6501	57.95
10	Fact	40.00	10.00	40.00	80.8247	78.83
11	Fact	40.00	10.00	40.00	75.4203	78.83
12	Fact	40.00	10.00	40.00	71.8975	78.83
13	Fact	30.00	8.00	100.00	56.1044	52.03
14	Fact	30.00	8.00	100.00	53.952	52.03
15	Fact	30.00	8.00	100.00	54.2343	52.03
16	Fact	40.00	8.00	100.00	72.4065	70.70
17	Fact	40.00	8.00	100.00	72.7946	70.70
18	Fact	40.00	8.00	100.00	64.2555	70.70
19	Fact	30.00	10.00	100.00	18.8426	19.66
20	Fact	30.00	10.00	100.00	17.0783	19.66
21	Fact	30.00	10.00	100.00	17.0783	19.66
22	Fact	40.00	10.00	100.00	55.8927	51.66
23	Fact	40.00	10.00	100.00	48.2357	51.66
24	Fact	40.00	10.00	100.00	49.259	51.66
25	Axial	23.93	9.00	70.00	9.2533	12.12
26	Axial	46.07	9.00	70.00	58.8782	55.90
27	Axial	35.00	6.79	70.00	81.4577	87.33
28	Axial	35.00	11.21	70.00	55.5556	49.57
29	Axial	35.00	9.00	3.60	66.2852	66.99
30	Axial	35.00	9.00	136.40	14.5161	13.69
31	Center	35.00	9.00	70.00	84.9946	81.02
32	Center	35.00	9.00	70.00	77.492	81.02
33	Center	35.00	9.00	70.00	83.1726	81.02
34	Center	35.00	9.00	70.00	80.0643	81.02
35	Center	35.00	9.00	70.00	79.064	81.02
36	Center	35.00	9.00	70.00	81.5291	81.02

For statistical analysis, a quadratic polynomial equation by central composite design was developed to predict the response as a function of independent variables and their interaction. In general, the response for the quadratic polynomials is described below:

1 $$Y=\beta_{0}+\Sigma \beta ixi+\Sigma \beta iixi_{2}+\Sigma \;\Sigma \beta ijxijxj$$

where *Y* is the response (dye decolorization); *β*_0_ is the intercept coefficient, *βi* is the linear terms, *βii* is the squared terms and *βij* is the interaction terms, and *xi* and *xj* are the uncoded independent variables (Ghadge and Raheman 2006). Data from the central composite experimental design were subjected to regression analysis using least square regression methodology to obtain the parameters of the mathematical models. The *F* test was then used to evaluate the significance of the model equation and model terms. The statistical significance of the model was evaluated using the analysis of variance (ANOVA) while, the optimal values were obtained by solving the regression equation and analyzing the response surface plot.

### Analytical methods

Residual dye in the original and treated samples was measured by monitoring the absorbance of the samples at 587 nm wavelength using a UV–visible double beam spectrophotometer (Shimadzu 160A Japan). Withdrawn samples were centrifuged at 12,000 rpm for 10 min to remove suspended particles and cells that may obstruct absorbance readings. The supernatants obtained after centrifugation were analyzed by measuring the decrease in absorbance at the wavelengths relevant to each dye with reference to the un-inoculated controls. Un-inoculated dye-free medium was used as blank. All assays were performed in triplicate and the average values were used in calculations. The extent of decolorization was calculated accordingly as follows:

2 $$\text{Decolorization}\;\left(\%\right)\;=\;\frac{\left(Ab_{0}-Ab_{1}\right)}{Ab_{0}}\times \;\text{1}00$$

where *Ab*_0_ is the absorbance of the dye solution before decolorization and *Ab*_1_ is the absorbance of the dye solution after decolorization. Microbial growth was monitored by checking the optical density of withdrawn samples before centrifugation. The optical density was studied at 660 nm after homogenously mixing samples.

### High performance liquid chromatography

Metabolic intermediates generated after bacterial decolorization and degradation of Direct Blue 71 were analyzed by HPLC. The HPLC (Agilent Technologies, USA) was equipped with a quaternary gradient pump system and a multi-wavelength detector. The mobile phase consisted of HPLC grade methanol and water in the ratio of 70:30. The samples were filtered with a 0.2-μm membrane filter before injection and 10 μL of the filtered samples was injected into the HPLC. The samples were eluted in gradient mode using a C18 column (Agilent ZORBAX, ODS 5 μm) at a flow rate of 1.0 mL/min at room temperature for 15 min.

### Determination of total organic carbon

The TOC content of the dye solutions was monitored during treatment under microaerophilic conditions and after agitation using a TOC analyzer (Shimadzu 5000A) with direct injection of the samples after centrifugation and filtration through a glass fiber filter (Whatman, USA). Data obtained were then used to calculate dye mineralization quantitatively, determined as TOC removal ratio, before and after the treatment process (Saratale et al. 2010):

3 $$\text{TOC removal ratio}\;\left(\%\right)\;=\;\frac{{\text{Initial TOC}}_{\left(\text{zero\textbackslash{}, h}\right)}-{\;\text{Observed TOC}}_{\left(t\right)}}{{\text{Initial TOC}}_{\left(\text{zero\textbackslash{}, h}\right)}}\times 100$$

where, TOC_(zero h)_ and TOC_(*t*)_ are the initial TOC value (at zero h) and the TOC value after a particular reaction time (*t*), respectively.

### Fourier transform infrared spectroscopy

FTIR analysis was used to investigate the changes in surface functional groups of the samples, before and after microbial decolorization. The analysis was carried out using Perkin Elmer 783 Spectrophotometer (Nicolet Analytical Instruments, Madison, WI). Liquid samples were loaded on the aperture of the liquid analyzer and the changes in the percent (%) transmission at different wavelengths were observed for treated samples and compared with control dye in the mid-IR region of 400–4,000 cm^−1^ with 16 scan speeds. The resolution of the spectrometer was 4 cm^−1^. The spectra were then subjected to baseline correction and the bands studied to quantify the changes in the chemical structure of the dye.

### Nuclear magnetic resonance spectroscopy

Proton nuclear magnetic resonance (1H NMR) studies were used for dye solutions before microbial treatment and for the degradation by-products. All 1H NMR spectra were recorded on a Bruker Avance 400 MHz spectrometer at 500.13 MHz at 295.3 K. Samples, dried by the classical double-pulsed field gradient of echo sequence WATERGATE, were dissolved in D_2_O, transferred to NMR tubes and the 1H spectrum recorded to observe the structural transformation in the dye molecules during treatment. A total of 32 scans were collected (acquisition time, 3.17 s; spectral window of 10,330.578 Hz). A 0.3 Hz line broadening was applied before Fourier transformation and a baseline correction of spectra were carried out prior to spectra integration with Bruker software.

## Results and discussion

### Decolorization profile of *P. aeruginosa*

**Fig. 2:**
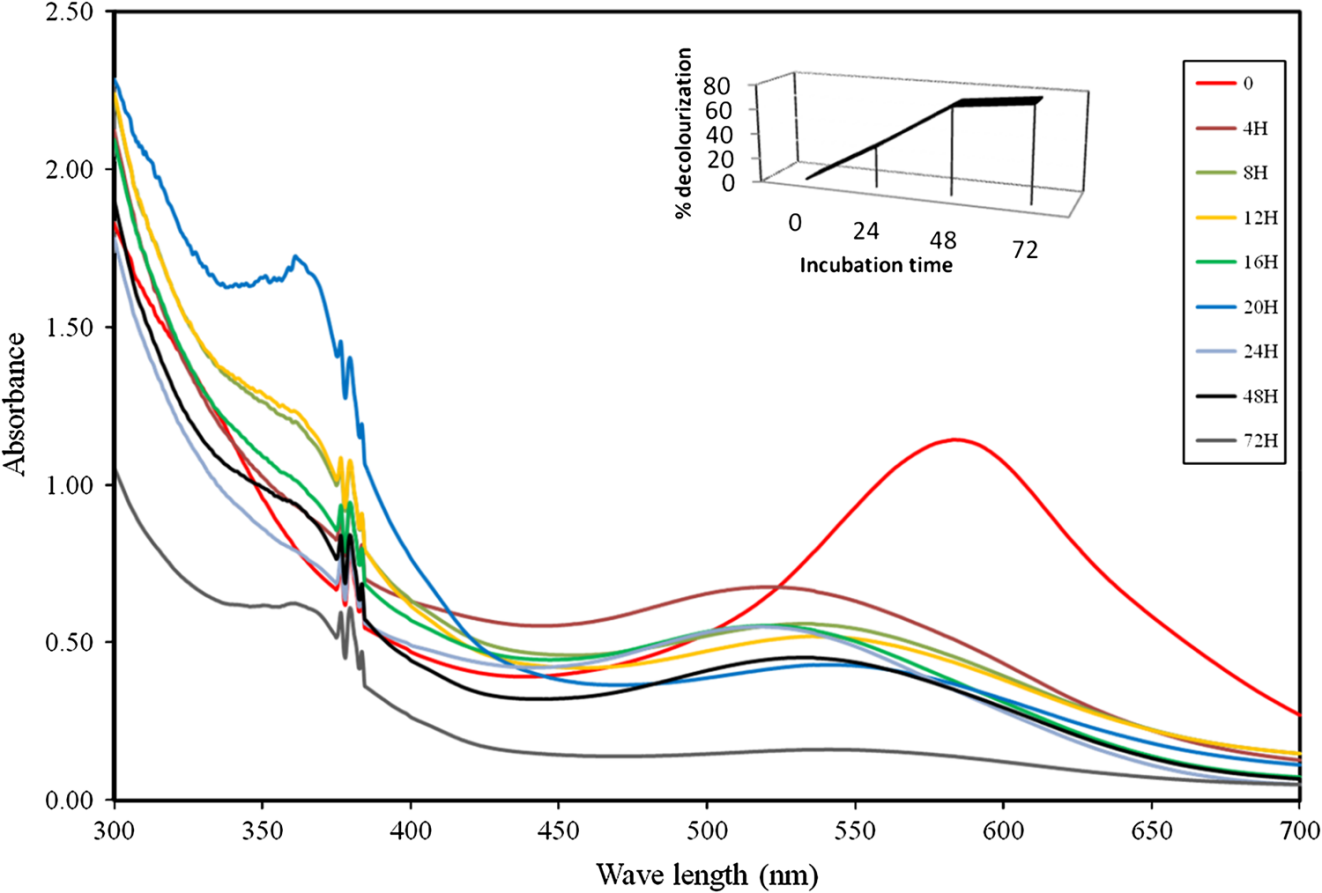
UV-visible spectral signatures of Direct Blue 71 showing the decolorization profile at various time intervals during dye degradation by *P. aeruginosa* under microaerophilic condition

Dye decolorization potential of *P. aeruginosa* was studied by growing the bacterium in BHM containing 50 mg/L of Direct Blue 71 at 30 °C under static condition. Data obtained showed that *P. aeruginosa* was able to effectively decolorize (70.43 %) Direct Blue 71 in solution within 48 h. The stepwise decrease in absorbance of dye with increase in time during the microaerophilic process is indicated on the time-dependent UV–visible spectra of withdrawn samples (Fig. 2). The Direct Blue 71 spectrum in visible region exhibits a main peak with a maximum absorbance at 587 nm. The decrease in the absorbance peak of the dye with time was indicative of the decolorization of the dye and cleavage of its azo bonds by azoreductases.

According to Chen et al. (2008), the decolorization of dyes by microorganisms can be attributed to adsorption to biomass or to biodegradation. However, in dye removal attributed only to biodegradation, either the major visible light absorbance peak will disappear or a new peak will appear. As shown in Fig. 2, the main absorbance peak of Direct Blue 71 almost disappeared within 48 h. New peaks observed may be due to light absorption by metabolites or degraded fragments of the dye molecules (Daneshvar et al. 2007; Khosravi et al. 2012), which indicated biodegradation.

Color change was slight in dye-containing medium when the experiment was conducted strictly under aerobic conditions albeit cell proliferation occurred faster due to sufficient dissolved oxygen. Chen (2002) had studied bacterial decolorization of azo dyes and considered it a non growth-associated entity. Earlier, it was reported that decolorization of azo dyes by *Proteus mirabilis* showed 20 % of color removal in shake culture but more than 95 % dye removal was estimated in static anoxic culture, even when associated with low level of cell growth than shaking condition (Chen et al. 1999). Decolorization of the dye under static culture conditions may be attributed to oxygen depletion and the subsequent creation of a microaerophilic environment in the flask for reduction of azo dye by the bacterial cells. Enhanced decolorization obtained under microaerophilic conditions when compared to data obtained for decolorization under aerobic conditions (data not shown) suggest that azoreductase played an important role in the cleavage of the azo bond (N=N) thus, resulting in generation of aromatic amines (Pinheiro et al. 2004; Pandey et al. 2007). Previous studies had provided evidence to show that microbial anaerobic azo reduction was linked to the electron transport chain, and suggested that dissimilatory azo reduction was a form of microbial anaerobic respiration (Hong et al. 2007). In an earlier report, the negative effect of agitation rate on decolorization activity was consistent with the increase in DO level and could be ascribed to the inhibition of azo reductase enzyme activity by oxygen (Chen and Lin 2007). In another study, decolorization of Direct Brown MR was observed effectively (91.3 %) in static anoxic condition even though at lower growth rate, whereas, agitated cultures grew well but showed less decolorization (59.3 %) within 48 h of incubation (Ghodake et al. 2009). Seesuriyachan et al. (2007) had also stated that the specific decolorization rate of another azo dye, Methyl Orange, was shown to be higher in reaction mixture under microaerophilic conditions rather than under aerobic condition.

No changes in color were obtained in the controls and in filter-sterilized microaerophilic-treated cultures suggesting that the elimination of color was not due to abiotic factors but rather due to the activities of the microorganism.

### Analyses of biodegradation metabolites formed

#### HPLC analysis

**Fig. 3:**
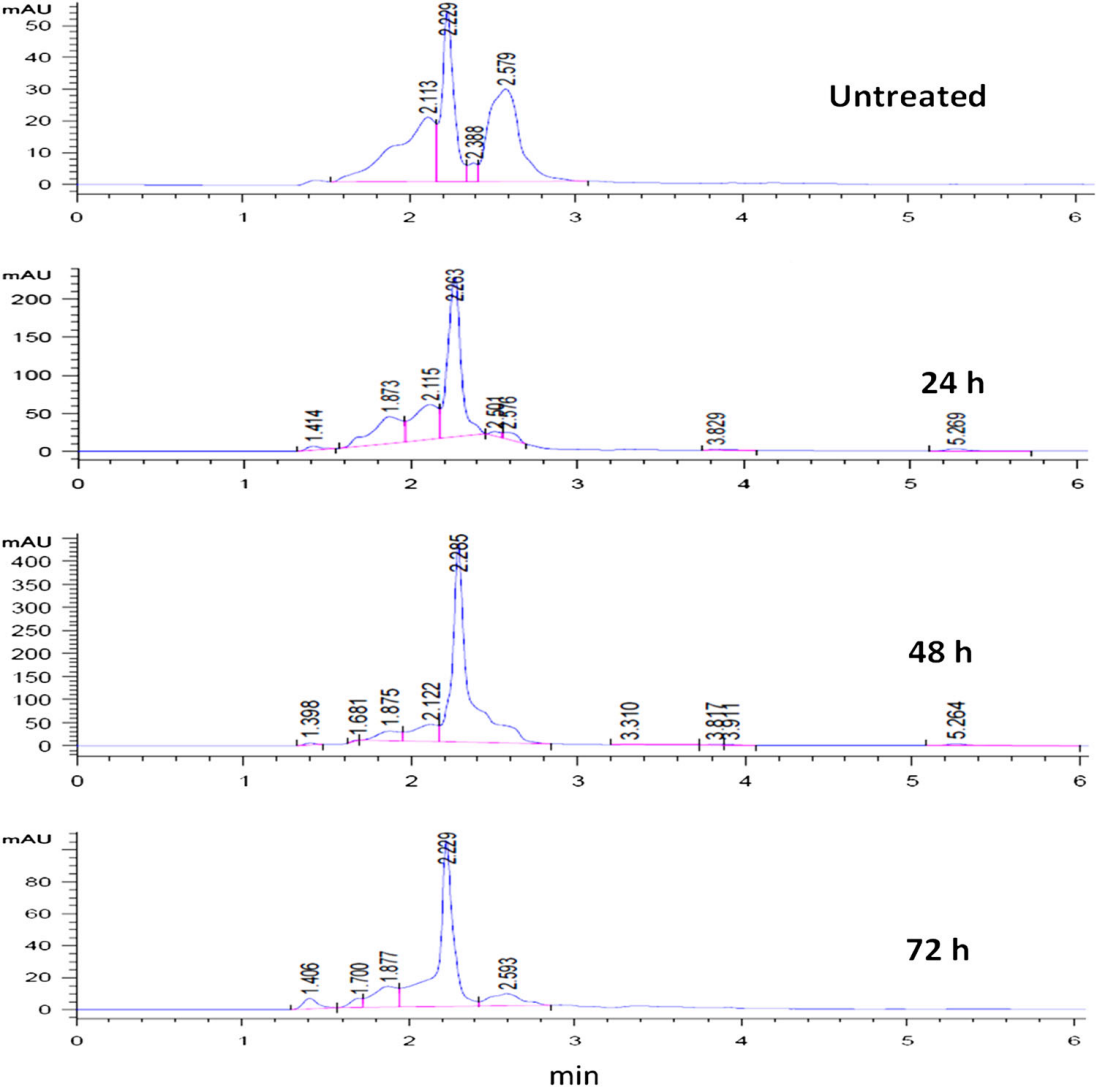
HPLC chromatograms of **a** Direct Blue 71 (control) and metabolic intermediates obtained after sequential **b** microaerophilic and **c** aerobic phase degradation by *P. aeruginosa*

The HPLC analysis of treated colored broth revealed that there was significant degradation of Direct Blue 71 during the microaerophilic and aerobic stages. The HPLC chromatogram (Fig. 3) shows a prominent peak (retention time; 2.229 min) in the untreated dye broth which may be attributed to a residual by-product (aromatic amine) of dye manufacture. This compound, with peaks at retention times of 2.263 and 2.285 min in 24 and 48 h spectra, respectively, increased in solution with time during microaerophilic treatment thus, indicating aromatic amine generation as a result of azo dye reduction. However, after aerobic treatment for 24 h, the peak decreased sharply suggesting the mineralization of the generated aromatic amines during aerobic treatment with *P. aeruginosa*. Likewise, peaks at retention times of 2.115, 2.579 min and beyond 3 min disappeared after aerobic treatment with the bacterium. This shows that the dye degradation metabolites produced under static conditions were removed in subsequent aerobic phase by *P. aeruginosa*.

#### TOC analysis

**Fig. 4:**
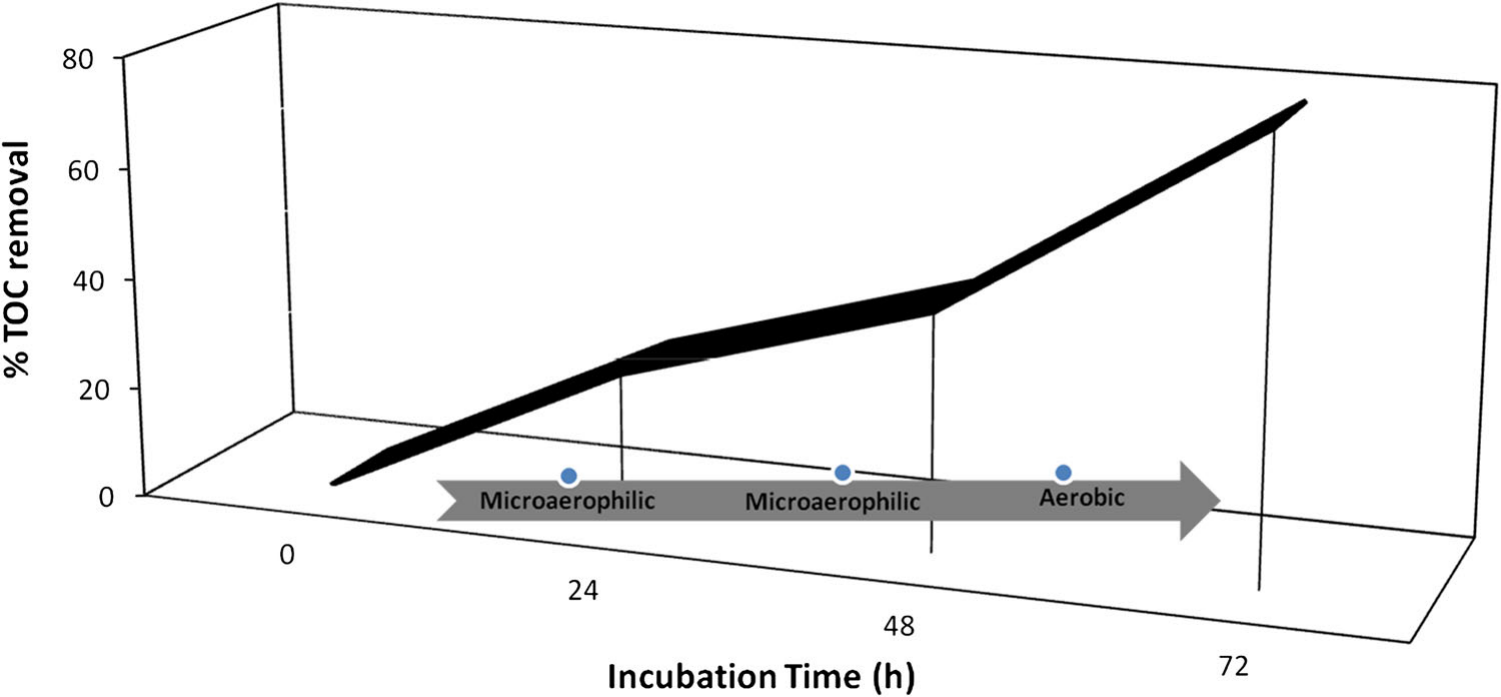
TOC removal during sequential microaerophilic/aerobic phase degradation of Direct Blue 71 by *P. aeruginosa*

Experiments carried out to determine changes in TOC during degradation of Direct Blue 71 by *P. aeruginosa* are as presented in Fig. 4. Reduction in %TOC was 26.31 and 42.58 after 24 and 48 h of incubation, respectively, under microaerophilic condition. However, subsequent incubation for 24 h under aerobic conditions resulted in 78.39 % reduction in TOC thus, suggesting the substantial conversion of the azo dye to CO_2_ by *P. aeruginosa* under these conditions. The switch to aerobic condition after 48 h of reduced oxygen concentration was to encourage the degradation of resultant decolorization metabolites (aromatic amines) which can only be mineralized under aerobic condition (Khosravi et al. 2013; Ogugbue et al. 2012a). No changes in TOC levels were obtained in the controls and in filter-sterilized microaerophilic-treated cultures. *P. aeruginosa* showed good potential for TOC removal compared to previously reported physicochemical methods viz. Fenton/UV-C process for Reactive Black 5 (Lucas and Peres 2007) in which a TOC removal of 46.4 % was obtained.

#### FTIR analysis

**Fig. 5:**
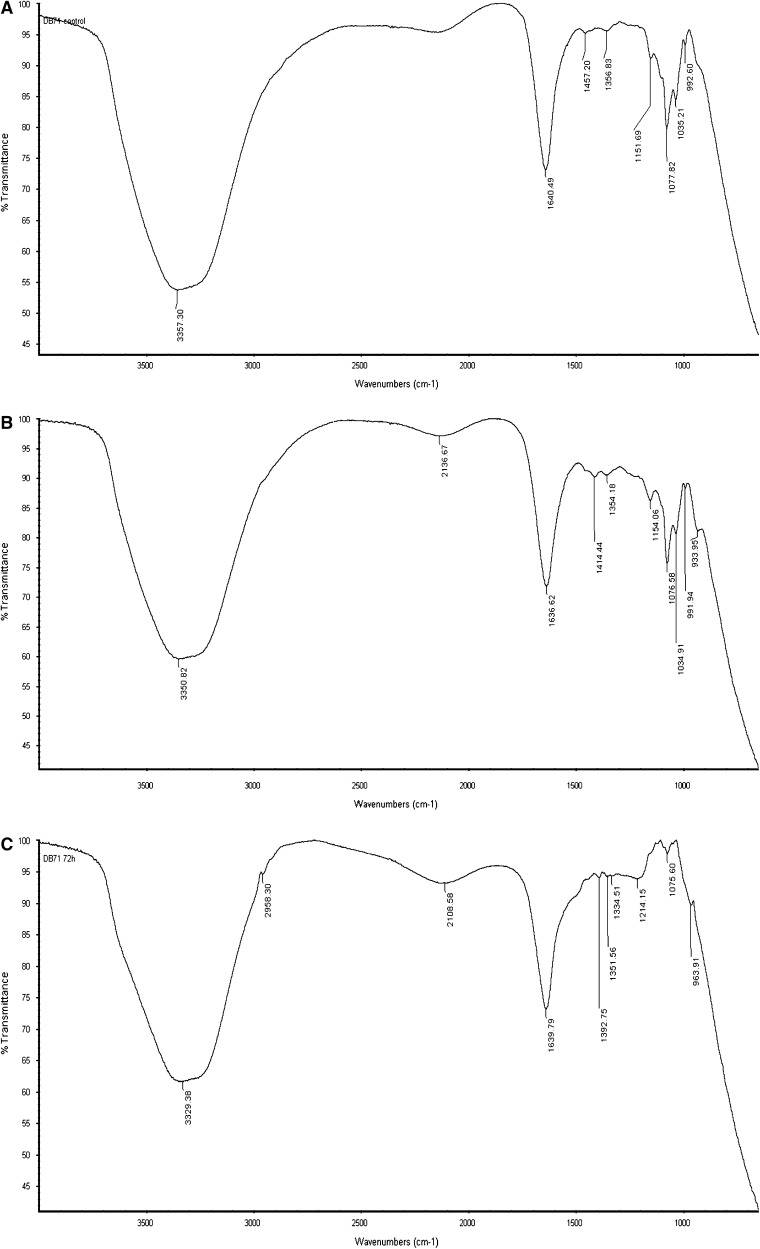
FTIR spectrum of **a** Direct Blue 71 (control) and its metabolites obtained after **b** microaerophilic phase and **c** aerobic phase treatments

The FT-IR spectra (Fig. 5a) obtained from the untreated dye samples showed several peaks in the region around 850 and 1,000 cm^−1^ usually associated with the out-of-plane bending vibration of substituted benzenes. After the microaerophilic and aerobic treatments, a significant reduction in absorption was observed in this region (Fig. 5b, c). Most of the absorption peaks observed in the parental Direct Blue 71 were either shifted or disappeared during decolorization. The peak at 1,154 cm^−1^ that could be attributed to acetates, formates, propionates and benzoates disappeared during the aerobic stage after reductive treatment thus, suggesting the biodegradation of these products formed during the reductive dye degradation. The band characteristic of azo bond in the region of 1,400–1,500 cm^−1^ of the dye (a) was absent in the biodecolorized samples (b, c) indicating the breakdown of –N=N– leading to color removal. A previous report had shown that the peak observed at 1,500 cm^−1^ characteristic due to azo bond (–N=N–) and the benzene ring at 1,400 to 1,600 cm^−1^ decreased with time (Hu et al. 2003). In the aerated samples, partial mineralization was suggested by the new peak observed at 2,958 cm^−1^, associated probably with C–H aliphatic stretching. During microaerophilic treatment, bands located within the region of 1,340–1,020 cm^−1^ stretching representing amine groups within the dye structure were retained but disappeared from the spectrum of the oxic treated dye broth. Kudlich et al. (1999) had reported that a large fraction of the aromatic amines from azo dyes are susceptible to autoxidation on exposure to air, producing water-soluble, highly colored dimers, oligomers and eventually dark-colored polymers with low solubility. In another study, an initially clear cultured medium of reduction products quickly turned slightly deep blue after changing to aerobic phase due to autoxidation of reduction products formed from anaerobic reduction of parent azo dyes (Supaka et al. 2004). However, in this study, no increase in any color shade was observed during the aerobic treatment suggesting that degradation of aromatic amines was probably due to microbial degradation rather than autoxidation.

#### NMR analysis

**Fig. 6:**
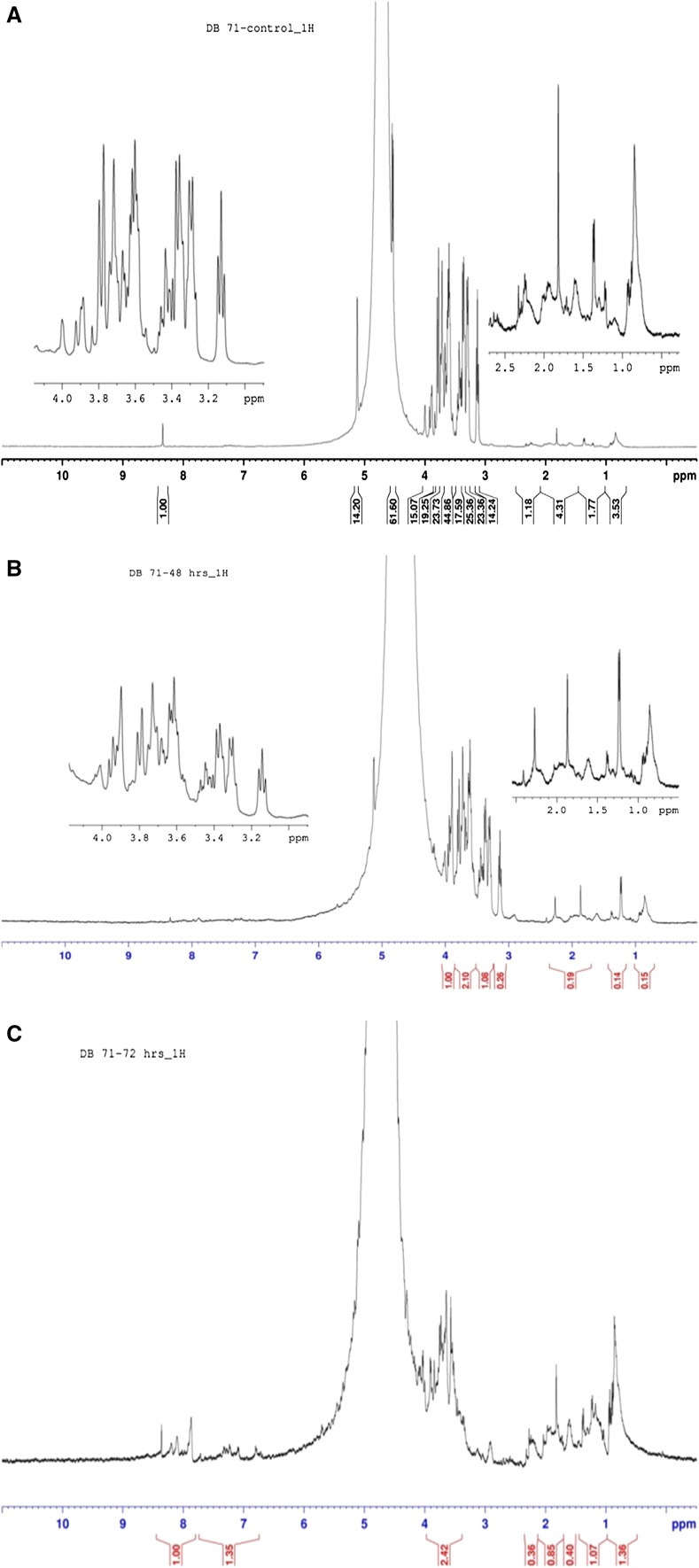
1H NMR data for Direct Blue 71 before and after degradation by *P. aeruginosa***a** untreated dye **b** after degradation under microaerophilic conditions **c** after degradation under aerobic conditions

The 1H NMR spectra of Direct Blue 71 before and after degradation by *P. aeruginosa* are shown in Fig. 6a–c. In control samples, the spectrum showed signals between 3.0 and 4.0 ppm corresponding to the protons of the aromatic rings. After treatment of dye solution under microaerophilic condition resulting in decolorization, no significant changes were observed in these peaks. This probably indicates the retainership of structural aromaticity even after azo bond cleavage. However, after treatment with the isolate under aerobic conditions, new signals appeared in the high-field region (between 0.5–2.0 ppm) indicating the formation of hydrocarbon aliphatic compounds. The spectrum peaks became attenuated in the aromatic region (3.0–4.0 ppm) with many of the major peaks resolved indicating loss of aromaticity and the biodegradation of this azo dye by *P. aeruginosa.* Data from the 1H NMR correlate with the UV/visible spectrophotometry and TOC data for degradation of the dye and aromatic amine intermediates after microaerophilic and aerobic phase treatments, respectively.

### Effect of process parameters

**Fig. 7:**
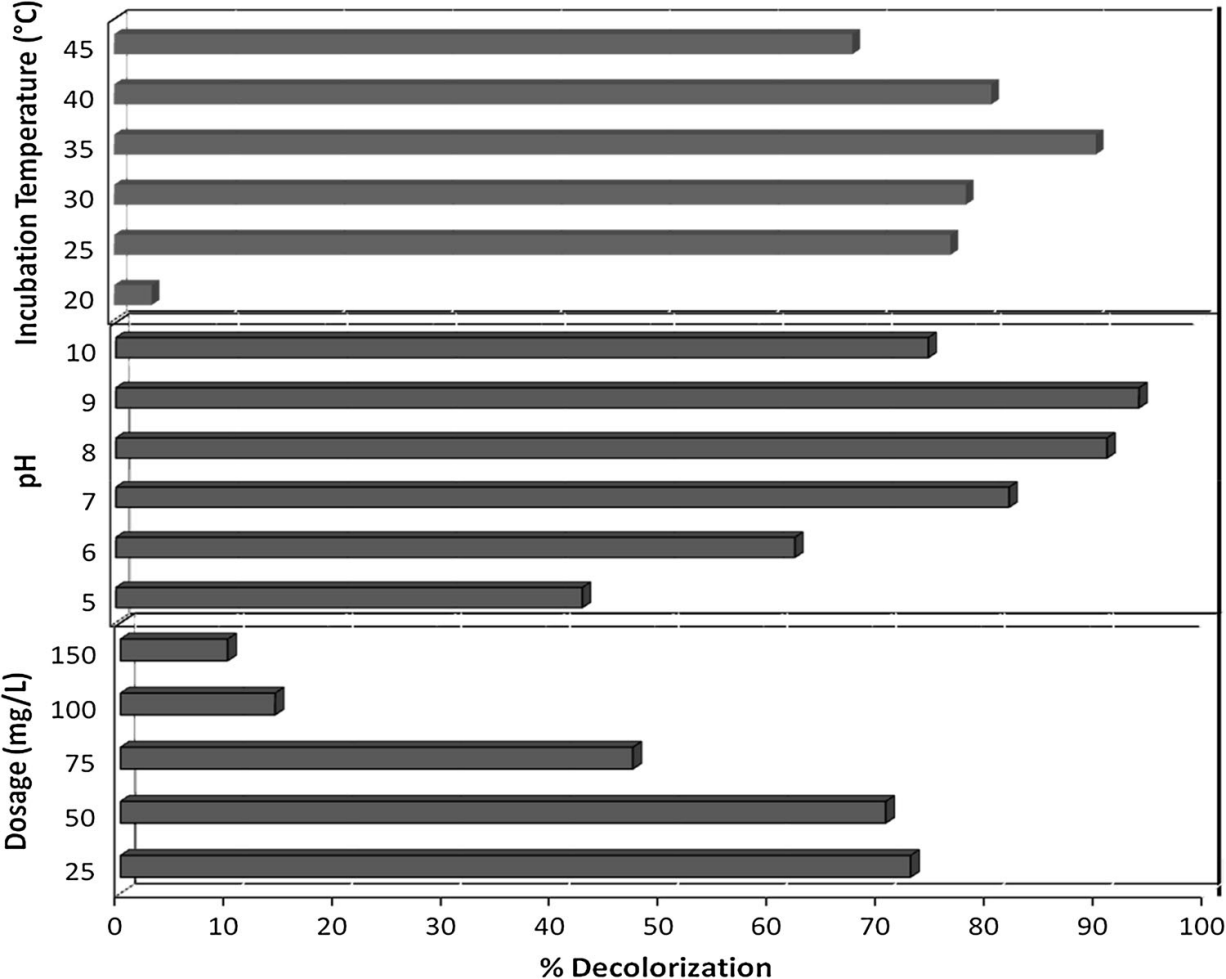
Effect of process parameters (incubation temperature, pH, dosage) on decolorization of Direct Blue 71 by *P. aeruginosa* under microaerophilic culture conditions (incubation time, 48 h)

Data obtained on effect of three process parameters on decolorization potential of *P. aeruginosa* are as presented in Fig. 7. The optimum temperature for decolorization (90.48 %) of Direct Blue 71 by *P. aeruginosa* was 35 °C after 48 h of incubation, though significant decolorization (above 75 %) was obtained at temperatures between 25 and 40 °C. At all temperatures, a greater extent of the color change was obtained within the initial 24 h of incubation. There was a 12 % increase in decolorization with an increase in incubation temperature from 30 to 35 °C suggesting the profound effect this parameter could have on dye removal capability of microorganisms. No significant decolorization was obtained at temperature below 25 °C or above 40 °C.

Decolorization experiments were carried out at a pH range of 5–10 in BHM while keeping other parameters constant. Effective decolorization (above 65 %) was obtained after 48 h of incubation in medium of pH, 6–10 with pH 8 being the optimum pH for decolorization (97.10 %) of Direct Blue 71 by *P. aeruginosa*. Decolorization was more than 40 % at all pH conditions studied however, drastic reduction in decolorization was obtained at pH below 5 or above 10.

Various initial dye concentrations (25–150 mg/L) were used to determine the maximum concentration that is non inhibitory to decolorization by *P. aeruginosa*. Data obtained showed that effective biodecolorization was obtained at concentrations up to 150 mg/L especially on prolonged incubation up to 144 h in BHM (pH, 7) at 30 °C. However, maximum decolorization (70.4 %) was obtained at 50 mg/L after 48 h of incubation. Beyond 150 mg/L, the extent of decolorization decreased significantly probably due to toxicity of the dye to the microbial cells due to increased concentration. This may also account for the initial (0–48 h) drag in decolorization rate at higher concentrations (75–150 mg/L) as cells were trying to get acclimatized to the high concentrations before maximum activity. Based on data obtained, temperatures of 30–40 °C and pH range of 8–10 were selected as the study range of interest for first (A) and second (B) factors whereas, a dosage range of 40–100 mg/L was used for factor C in the optimization study. These results were used to plan and carry out a systematic study of the dye decolorization process through the central composite design. The main objective was to determine the optimal operational conditions for the variables that would ensure decolorization of Direct Blue 71 within 24 h of incubation by *P. aeruginosa* or to determine a region that satisfied the operating specifications (Ravikumar et al. 2006).

### Process optimization using RSM

A total of 36 experiments with different combinations of temperature (factor A, 30–40 °C), pH (factor B, 8–10) and dosage (factor C, 40–100 mg/L) were performed within 24 h using the CCD method. Dye decolorization was used as the response (*Y*, %) and the results obtained from the experiments (observed and predicted) are summarized in Table 1. By applying multiple regression analysis, the following second-order polynomial equation (in coded units) that could relate dye decolorization to the parameters studied was obtained as Eq. (4):

4 $$Y=81.02+9.89A-8.53B-12.04C-9.60A^{2}-2.57B^{2}-8.30C^{2}+3.33AB+2.78AC-4.32BC$$

From the Eq. (4) above, the second-order response functions are represented by: *Y*, the response for dye decolorization; *A*, the coded value of variable incubation temperature; *B*, the coded value of variable pH and *C*, the coded value of variable dye concentration (dosage). Data obtained on predicted values and observed values show the empirical models actually fit the actual data with *R*^2^ of 0.9624. When *R*^2^ is closer to unity as obtained in this study, the empirical models fit the actual data better whereas, the relevance of the dependent variables in the model in explaining the behavior of variations cannot be ascertained by a smaller value of *R*^2^ (Cao et al. 2008).

Table 2 shows the data obtained from the analysis of variance (ANOVA) for the response surface reduced quadratic model. The model *F*-value is 73.93. The value of “Prob > *F*” for the models is less than 0.05 to indicate that it is significant and desirable as it shows that the terms in the model have a significant effect on the response. The value of *P* < 0.0001 indicates that there is only a 0.01 % chance that a “model *F*-value” could occur due to noise in the experiment. Values of “Prob > *F*” less than 0.05 indicate model terms are significant. In this case A, B, C, A^2^, B^2^, C^2^, AB, AC, BC are significant model terms. A value greater than 0.05, would have indicated that the model terms are not significant.

**Table 2:** ANOVA analyses for the response surface quadratic model

Source	SS	DF	MS	*F* value	Prob > *F*	
Model	16,372.74	9	1,819.19	73.93	<0.0001	Significant
A	3,305.71	1	3,305.71	134.34	<0.0001	
B	2,458.58	1	2,458.58	99.91	<0.0001	
C	4,900.86	1	4,900.86	199.17	<0.0001	
A^2^	3,369.60	1	3,369.60	136.94	<0.0001	
B^2^	240.85	1	240.85	9.79	0.0043	
C^2^	2,522.61	1	2,522.61	102.52	<0.0001	
AB	266.41	1	266.41	10.83	0.0029	
AC	185.32	1	185.32	7.53	0.0108	
BC	448.85	1	448.85	18.24	0.0002	
Residual	639.78	26	24.61			
Lack of fit	163.41	5	32.68	1.44	0.2510	Not significant
Pure error	476.37	21	22.68			
Cor total	17,012.52	35				
Std. Dev.	4.96	*R*-squared				0.9624
Mean	61.80	Adj *R*-squared				0.9494
C.V.	8.03	Pred *R*-squared				0.9171
PRESS	1,410.47	Adeq precision				28.767

*SS* Sum of squares, *DF* degree of freedom, *MS* mean of squares

The “Lack of Fit *F*-value” of 1.44 implies that the Lack of Fit is not significantly relative to the pure error. There is a 25.10 % chance that a “Lack of Fit *F*-value” this large could occur due to noise. Insignificant lack of fit is considered as a good indication that the model can fit. The “Pred *R*-Squared” of 0.9171 is in reasonable agreement with the “Adj *R*-Squared” of 0.9494. “Adeq Precision” measures the signal to noise ratio. A ratio greater than 4 is desirable. The ratio of 28.767 indicates an adequate signal indicating that this model can be used to navigate the design space.

**Fig. 8:**
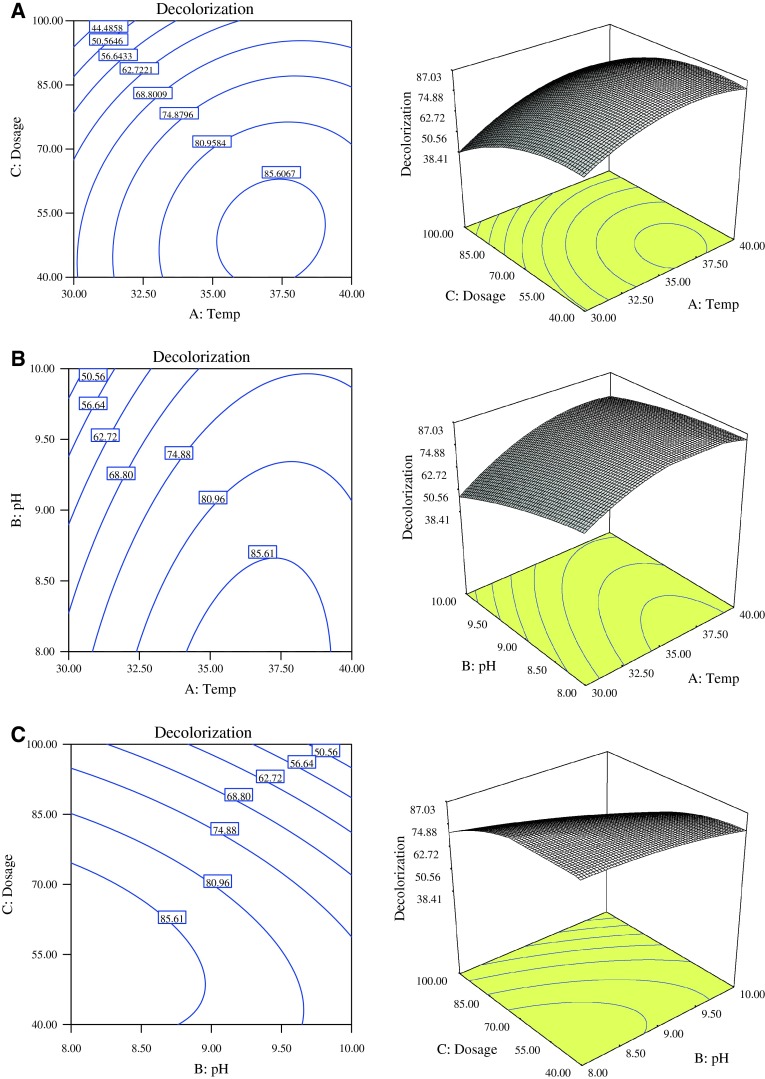
**a** Contour plot and 3-D surface showing dye decolorization extent as a function of temperature and dye concentration (Actual factor B: pH = 8.02). **b** Contour plot and 3-D surface showing dye decolorization extent as a function of temperature and pH (Actual factor C: dye concentration = 49.95 mg/L). **c** Contour plot and 3-D surface showing dye decolorization extent as a function of pH and dye concentration (Actual factor A: temperature = 35.15)

The choices for level combinations of the three variables such as, temperature, pH and dye concentration were made into contour plots (Fig. 8a–c) which indicated the percentages of decolorization within 24 h of incubation. The behavior of percentage decolorization with respect to changes in temperature and dye concentration is shown in Fig. 8a. These two parameters showed positive influence on dye decolorization. The percentage dye decolorization increased with increase in temperature and dosage until a certain level where further increases in both parameters led to a decrease in dye decolorization. In Fig. 8b, the variation of percentage decolorization as specified by the variables, pH and temperature shows that with the increase in pH, the percentage dye decolorization decreased. The result shows that optimal percentage dye decolorization was between 35 and 37 °C and slightly above neutral pH. Three dimensional and contour plots for interaction effect of reaction pH and dye concentration toward dye decolorization are shown in Fig. 8c. Dye decolorization increased as the pH increased to its medium level (pH 8) and dosage increased to its central level (70 mg/L). Optimal decolorization was obtained in culture broth of pH 8 and dye concentration, 50 mg/L. Generally, a stronger influence of pH and dye concentration occurred when both parameters were at their median level. The decreasing dye decolorization at higher concentrations was probably as a result of increasing toxicity of the dye to the microbial cells and possible enzyme inactivation at such high concentrations. Dyes have been reported to be inhibitors of nucleic acid synthesis and cell growth (Ogawa et al. 1989).

In each contour plot, the other one variable was held constant. Generally, increase in the three process parameters to a certain threshold value resulted in increase in percentage decolorization of Direct Blue 71 by *P. aeruginosa*. This is due to the positive quadratic model as shown in Eq. 4. It also indicates that the experimental value must consider the running effect of these significant factors at the stipulated levels to maximize dye decolorization by *P. aeruginosa*.

### Verification experiment

To verify the results obtained from the statistical analysis of CCD, five verification experiments were carried out under the optimal conditions obtained through RSM. Altogether, five solutions for optimal conditions were generated by the software according to the order of suitability. One of the solutions was then chosen for further process studies to confirm the validity of the statistical experimental strategies with experimental data. The optimal decolorization conditions were as follows: temperature, 35.15 °C; pH, 8.01 and dye concentration, 49.95 mg/L. The average decolorization rate achieved was 84.80 % within 24 h of incubation. The experimental value obtained for color removal was found to be quite close to the predicted value (88.99 %) using RSM as shown in Table 3. These results confirm the predictability of the model for color removal within the experimental conditions used. The use of RSM to optimize process parameters during dye decolorization experiments has been reported by other researchers (Mohana et al. 2008; Lau and Ismail 2010).

**Table 3:** Results of validation experiments conducted at optimum conditions as obtained from DOE

Solutions number	A	B	C	Predicted value (%)	Actual value (%)	Desirability
1	34.67	8.20	54.48	88.73	82.07	1.000
2	38.39	8.33	51.30	88.88	82.79	1.000
3	35.79	8.11	50.78	88.61	84.05	1.000
4	36.45	8.09	55.62	88.51	80.36	1.000
5	35.15	8.01	49.95	88.99	84.80	1.000 Selected

## Conclusion

In this study, the application of *P. aeruginosa* to the treatment of synthetic dye wastewater was investigated using RSM by varying control variables such as temperature, pH and dye concentration. The function of these variables in terms of dye decolorization was well explained by the results obtained from RSM and was used to optimize the conditions for maximum dye removal. By applying the RSM design to the optimization experiments, the process variables were well studied and decolorization extent up to 88 % was achieved in a shorter time (24 h). The experimental and the predicted values were very similar which reflected the accuracy and the applicability of RSM. A comparative UV–vis, HPLC, TOC, FTIR and NMR analyses revealed significant changes in peak positions and indicate the generation of intermediate products during degradation of Direct Blue 71 by the bacterium. Efficient color removal and mineralization of Direct Blue 71 by *P. aeruginosa* suggest its potential for real industrial applications in treatment of colored textile dyestuff effluents.

## Abbreviations

BOD: Biochemical oxygen demand
COD: Chemical oxygen demand
C.I.: Color index
CFU: Colony-forming units
OD: Optical density
UV: Ultra violet
*λ*_max_: Maximum absorption wavelength
3-D: 3-Dimensional
